# Impact of total splenectomy on peripheral lymphocytes and their subsets in patients with hypersplenism associated with cirrhotic portal hypertension

**DOI:** 10.1038/s41598-021-00692-x

**Published:** 2021-10-28

**Authors:** Yunfu Lv, Hongfei Wu, Wan Yee Lau, Jinfang Zheng, Jincai Wu, Min Zeng

**Affiliations:** 1 Department of General Surgery, Hainan General Hospital (Hainan Medical College Affiliated People’s Hospital), Haikou, 570311 China; 2 Faculty of Medicine, Chinese University of Hong Kong, Shatin, Hong Kong SAR China

**Keywords:** Cell biology, Immunology

## Abstract

To study the impact of total splenectomy (TS) on peripheral lymphocytes and their subsets in patients with hypersplenism associated with cirrhotic portal hypertension (CPH). We studied 102 consecutive patients who received TS from January 2008 to January 2020 due to CPH-related hypersplenism. A similar number of healthy individuals are used as healthy controls (HC). The total lymphocyte counts and their percentages of B lymphocytes, total T lymphocytes (cluster of differentiation (CD)3^+^) and their subsets (CD4^+^, CD8^+^), and natural killer (NK) cells in preoperative peripheral blood samples in hypersplenism patients were significantly lower than that of the HCs (both *P* < 0.05). The total lymphocyte counts and percentages of B lymphocytes in peripheral blood were significantly increased 1 week and 1 month after TS when compared with the pre-TS values (*P* < 0.05). There was no significant difference in the percentages of NK cells before or after surgery (*P* > 0.05). However, the percentages of CD3^+^ cells was significantly higher 1 month after than before surgery (*P* < 0.001). The percentages of CD4^+^, and CD8^+^ T lymphocytes were significantly lower 1 week after surgery (*P* < 0.05), but they were significantly higher 1 month after surgery (*P* < 0.01). The CD4^+^:CD8^+^ ratio was not significantly different from those before surgery, and 1 week or 1 month after surgery (*P* > 0.05). Patients with hypersplenism associated with CPH were significantly immunosuppressed preoperatively. After TS, the total lymphocyte count and percentages of B lymphocytes, and total T lymphocytes and their subsets increased significantly, resulting in improved immune functions.

## Introduction

Hypersplenism associated with cirrhotic portal hypertension is a common condition that is often complicated by peripheral cytopenias^1^.The more spectrum of peripheral blood cytopenia affects, the worse the prognosis, and may even endanger the life of the patient^2^. Peripheral cytopenias can be caused by hypersplenism, non-hypersplenism, and a combination of other factors, though hypersplenism is still the major contributing factor^3^.

The definition of hypersplenism was controversial until in 1955, Dameshek^4^ defined hypersplenism by presence of four conditions: (a) splenomegaly; (b) mono-lineage or multi-lineage cytopenias; (c) normal status or hyperplasia of bone marrow; (d) correction of cytopenias after splenectomy.Strictly speaking, it is not appropriate to correct hypocytopenia after splenectomy as one of the criteria for diagnosing hypersplenism. Because various shunts can also correct hypocytopenia, indicating that dredging venous blood flow is the key. It can be speculated that the retention of blood cells in the spleen may be the main reason for the reduction of peripheral blood cells. However, the lack of such reports in the literature is collectively referred to as hypersplenism. The ideal diagnosis of hypersplenism in cirrhosis and portal hypertension should have three: First of all, non-hypersplenism factors that cause peripheral cytopenias such as toxic effects of hepatitis viruses on bone marrow^5–7^, gastrointestinal bleeding^8^, Severe infection,immunocompromised status^9,10^, drug toxicity^11–13^, platelet destruction in peripheral circulation^14^, Blood disease, and hematopoiesis disorders caused by deficiency of vitamins and nutrients^2,15,16^ should be excluded. (1) The spleen enlarges, the blood storage function of the spleen and the function of destroying blood cells through phagocytosis, production of abnormal antibodies and immune imbalance are enhanced, resulting in an increase in the number of blood cells stored and destroyed in the spleen. (2) There is a decrease in one or more blood cell components in the blood. This diagnosis does not include splenectomy and shunt to eliminate the content of peripheral blood cell reduction because they have to wait for surgery to be diagnosed, and hypersplenism should be diagnosed before surgery or patients without surgery, and use it to guide treatment.

The spleen is the largest lymphoid organ. Lymphocytes in the spleen account for approximately 25% of all the lymphocytes in the body. The spleen forms an important part of the whole immune system. T lymphocytes, B lymphocytes, and natural killer (NK) cells are the main immune cells in the spleen. The immune cells and factors in the spleen undertake non-specific immune functions through phagocytosis, and they also carry out specific immune functions through cellular and humoral immunity mediated by T and B lymphocytes. The spleen also participates in the immune response by adjusting the proportion of immune cells within the spleen and in peripheral blood^17^. Lymphocytes are a type of immune cell in the circulation, and are composed of T and B lymphocytes, the latter being the main humoral immune cells. NK cells are the main effectors of innate immune response^18^, and they mediate innate immune responses. They do not rely on antibodies or complements, but can kill target cells directly, thus playing a part in resisting infection and immune regulation/surveillance^19,20^.

The total number of clusters of differentiation (CD)3^+^ cells reflects the total number of T lymphocytes which can be divided into two subsets, CD4^+^ and CD8^+^, according to their phenotypes and functions. The T-lymphocyte subsets are the most important cell populations in the immune system. CD4^+^ cells are mainly T helper (Th) cells which secrete interleukins, tumor necrosis factor and other cytokines, and express different surface molecules. They play an important part in regulation of specific and non-specific immunity, as well as cellular and humoral immunity^21^. CD8^+^ cells exert cytotoxicity to inhibit T lymphocytes^18^ and are the main effectors of the immune system to eliminate virus-infected cells^17^. Appropriate immune functions are maintained through the interaction of the T-lymphocyte subsets^1^. The CD4^+^:CD8^+^ ratio is an important indicator that reflects directly a balanced immune function of host T cells.

Most patients with cirrhosis and portal hypertension and hypersplenism can be treated non-surgically. However, a small number of patients require surgical treatment based on total splenectomy due to factors such as severe hypersplenism, repeated gastrointestinal bleeding, or a huge spleen that affects the quality of life, or poor results of non-surgical treatment.In the past, people did not know enough about the peripheral lymphocytes and their subgroups in patients with cirrhosis and portal hypertension and hypersplenism. In particular, there was almost no report on the impact on them after total splenectomy, so we conducted the research.

## Patients and methods

### The study cohort

Ethical approval for this research project was obtained from the Ethics Committee of Hainan General Hospital (Hainan, China) (Ethical Lot Number: Med-Eth-Re [2021] 003). Consecutive patients with hypersplenism associated with cirrhotic portal hypertension who underwent TS at Hainan General Hospital (Hainan, China) from January 2008 to January 2020 were enrolled in this study. These patients formed the “TS group”.

All methods were performed in accordance with the relevant guidelines and regulations/Declaration of Helsinki.Informed consent obtained from all patients in every research.

### The control cohort

During the study period, a volunteer group of the same number of healthy individuals matched with age and gender with the TS group was recruited to form the control group.

### Treatment

All patients in the TS group underwent total splenectomy as a part of the surgical treatment. The control group of individual did not receive any medical or surgical treatment, and they were completed healthy individuals.

### Blood taking for cell counts

In the TS group of patients, peripheral blood was taken preoperatively to determine the total lymphocyte count, the percentages of B lymphocytes, NK lymphocytes, total T cells (CD3^+^) and T-cell subsets (CD4^+^ and CD8^+^), as well as counts of white blood cells (WBCs), red blood cells (RBCs) and platelets. These tests were repeated 1 week and 1 month after surgery. In the control group of normal and healthy individuals, peripheral blood was taken for the same preoperative blood tests as in the TS group of patients.

### Statistical analyses

The collected data were entered into Epidata 3.1, analyzed using SPSS 26 (IBM, Armonk, NY, USA) and Rstudio 1.1.456 (R Project for Statistical Computing, Vienna, Austria), and visualized using Prism 8.2.1 (GraphPad, San Diego, CA, USA). Measurement data were expressed as mean ± standard deviation.Count data were expressed as percentages. A *P* < 0.05 was considered significant.

## Results

The TS group consisted of 102 patients, with 57 men and 45 women. The mean age +/− standard deviation was 43.6 ± 5.9 years. The underlying etiologies of liver cirrhosis were hepatitis B virus (HBV) infection in 61 patients (59.8%), hepatitis C virus (HCV) infection 15 patients (14.7%), alcoholic hepatitis 11 patients (10.8%), cholestasis 7 patients (6.9%) and other causes 8 patients (7.8%). Overall, 81 patients (79.4%) had Pugh-Child grade A cirrhosis and 21 (20.6%) had grade B cirrhosis. TS was carried out in these patients as a part of the operations for massive gastrointestinal bleeding of ≥ 500 ml (n = 54), a low platelet count of ≤ 5 × 109/L (n = 31) and splenomegaly (n = 17). In addition, 91 of these 102 patient underwent pericardial devascularization. There were 102 healthy individuals included into the control group which consisted of 58 males and 44 females, with a mean +/− standard deviation age of 44.13 ± 3.6 years. There were no significant differences between groups in age and gender (*P* > 0.05). No healthy individuals in the control group had any history of liver diseases and they had not received any medical or surgical treatment for any associated medical conditions.

**Figure 1:**
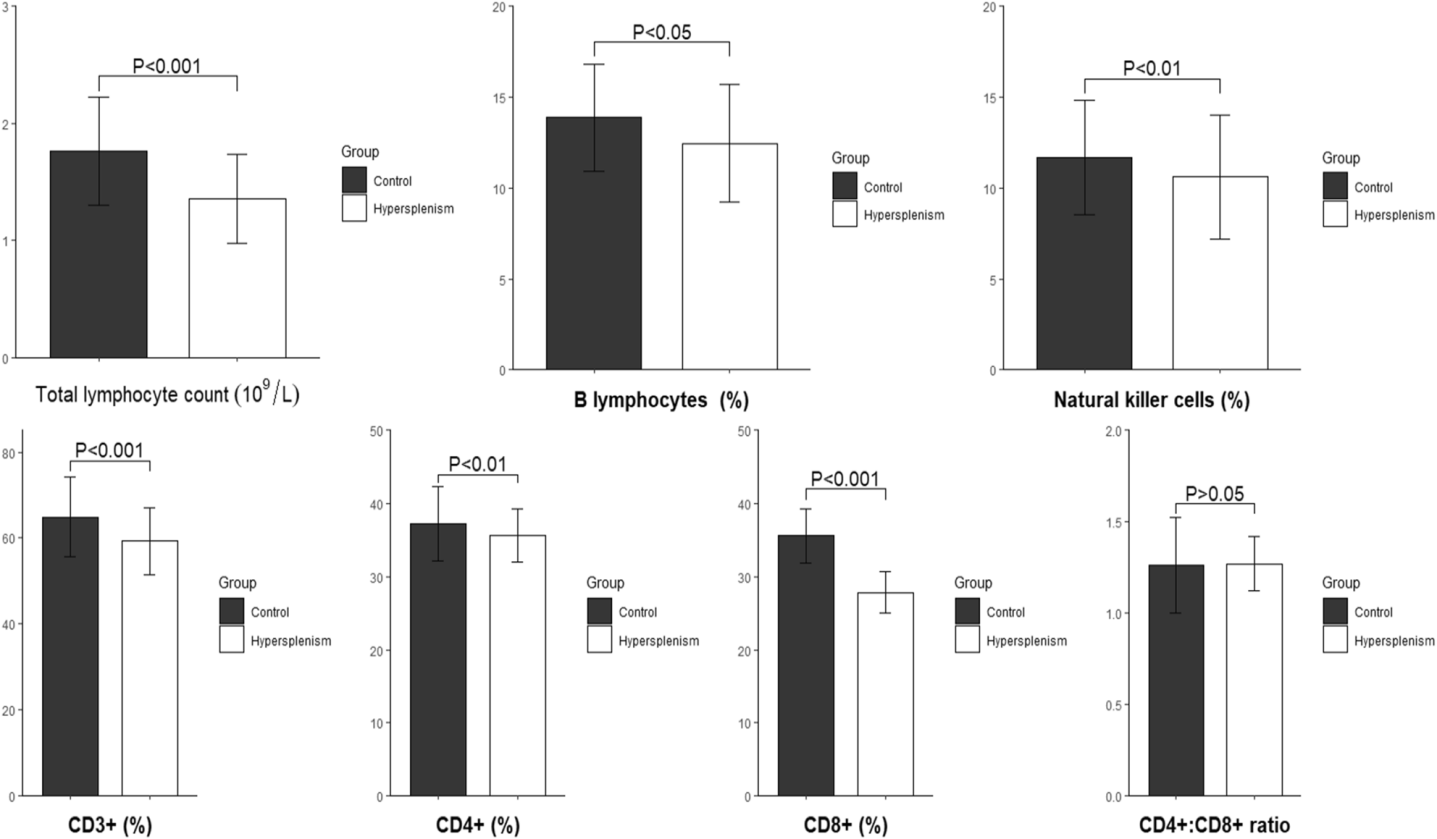
Comparison of peripheral lymphocytes and their subsets between the hypersplenism group and control group.

Before surgery, the total lymphocyte count and percentages of B lymphocytes and NK cells in peripheral blood were significantly lower in the TS group than those in the control group (*P* < 0.05). In the TS group, the total lymphocyte count and percentage of B lymphocytes in peripheral blood increased significantly 1 week and 1 month after surgery when compared with those before surgery (both *P* < 0.05) (Fig. 1). There was no significant difference in the percentage of NK cells before and after surgery (*P* > 0.05).

**Figure 2:**
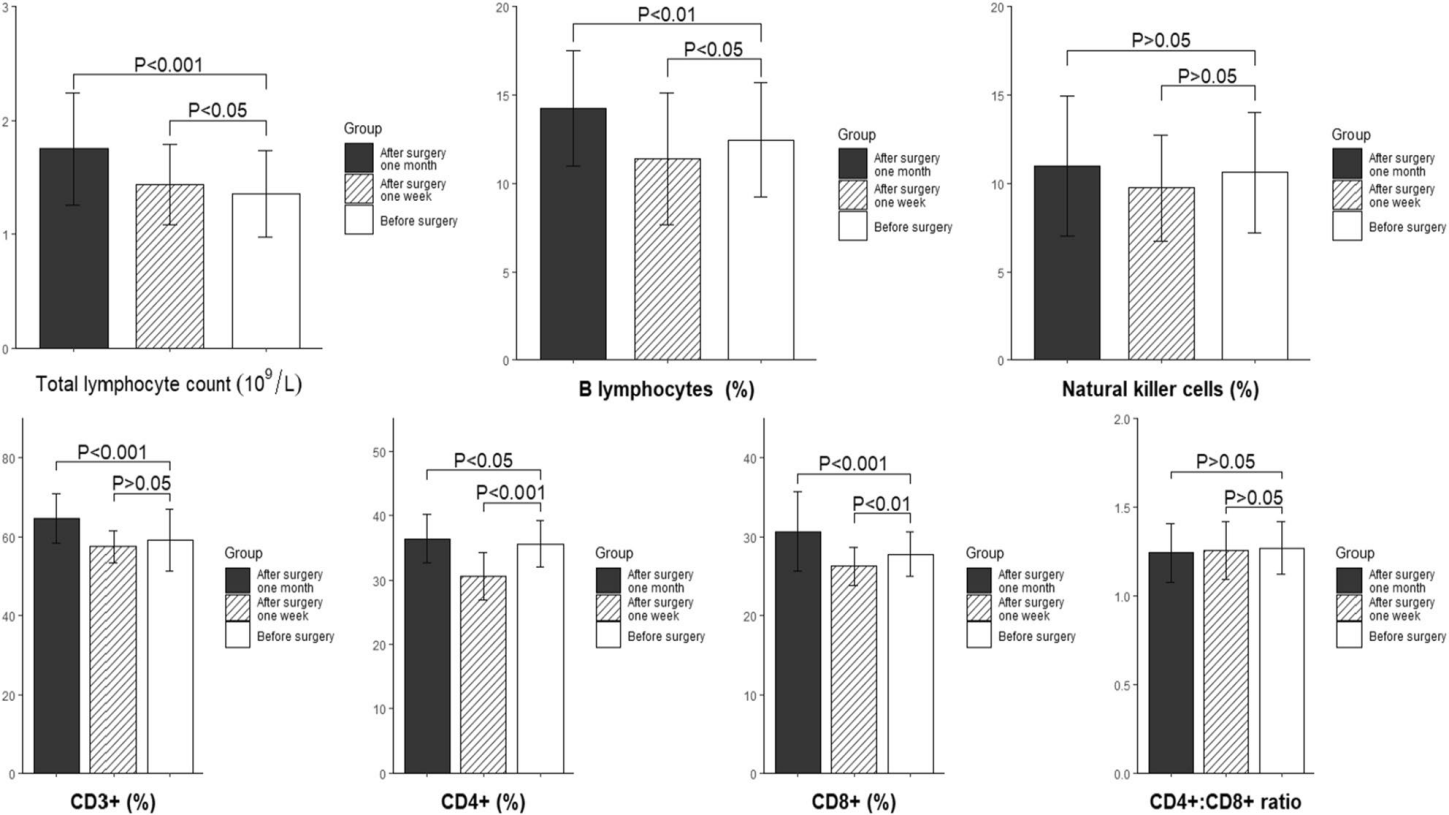
Changes in peripheral lymphocytes and their subsets in patients with hypersplenism before and after surgery.

Before surgery, the percentages of total T lymphocytes (CD3^+^) and their subsets (CD4^+^ and CD8^+^) in peripheral blood were significantly lower in the TS group than those in the control group (*P* < 0.01).In the TS group, when compared with the results before surgery, the percentage of CD3^+^ lymphocytes increased significantly 1 month after surgery (*P* < 0.001). In addition, the percentages of CD4^+^ and CD8^+^ lymphocytes were significantly lower 1 week after surgery (*P* < 0.05), but they were significantly higher 1 month after surgery (*P* < 0.01) (Fig. 2). The CD4^+^:CD8^+^ ratio showed no significant difference before or 1 month after surgery (*P* > 0.05).

## Discussion

Chu and colleagues^22^ stated that further research is required to ascertain if subtotal splenectomy can improve the immune function of the residual spleen in patients with cirrhotic splenomegaly. In the present study, the total lymphocyte count and the percentages of B lymphocytes, NK cells, and total T lymphocytes and their subsets in the peripheral blood in patients with hypersplenism associated with cirrhotic portal hypertension before surgery were significantly lower than those of the control group. The reduction in the number of lymphocytes and their subgroups suggested an immunocompromised status of these patients, with consequent impairment of liver function or aggravations of the existing liver dysfunction. Thrombopoietin production by hepatocytes^23–25^ is reduced as a result and ultimately can lead to a decrease in platelet counts in the peripheral circulation. The compromised or disordered immune functional status can also lead to the production of autoantibodies to blood cells, resulting in accelerated blood cells destruction^26^. The numbers of these cells and their subsets increase significantly to normal or above-normal levels after total splenectomy, which is similar to the relative increase in the number of T lymphocytes after splenectomy as reported by Graffner and coworkers^27^. The explanation for this phenomenon is likely to be related to hypersplenism as hypersplenism can result in decrease in WBCs, RBCs, and platelets in peripheral blood. Lymphocytes are a type of WBC, and any decrease in WBC count leads to a decrease in lymphocytes and their subsets. Increase in WBC count after total splenectomy with increase in lymphocytes and their subsets^28^, result in an improved immune functional status.

T lymphocytes play an important role in the immune system. However, if they are not activated, they cannot initiate signal transduction as mediated by cytokine receptors, nor can they activate the immune system^29^. There are approximately 94 genes involved in the activation of T lymphocytes^30^. Activated T lymphocytes produce various functional subsets, with CD4^+^ and CD8^+^ cells being the main subsets produced in the thymus. The CD4^+^:CD8^+^ ratio reflects cellular immune function and is an important measure of immune balance^18^. After CD4^+^ cells enter into peripheral immune organs, they are activated by a complex of antigen peptides and major histocompatibility complex (MHC) class-II molecules and they are called Th cells. After activation by a complex of antigen peptides and MHC class-I molecules, CD8^+^ cells can kill infected or otherwise harmed cells and they are called cytotoxic T cells^21^. In health, the CD4^+^:CD8^+^ ratio is relatively balanced, and the normal range is approximately 1.4–2.5^31,32^. In fact, there are no adverse effects if the ratio is > 1, or otherwise immune dysfunction occurs. In the present study, although the percentages of CD4^+^ and CD8^+^ cells increased significantly after splenectomy, the ratio remained > 1.2, and this did not result in any diseases caused by immune dysfunction.

Whether splenectomy is indicated for hypersplenism is controversial^33^. Total splenectomy can reduce symptoms caused by splenomegaly, including abdominal distension, pain, and a feeling of fullness^34^. It corrects hypersplenism, promotes recovery of WBCs, platelets and RBCs, reduces liver fibrosis, and improves liver function^35–37^. However, some researchers believe that splenectomy was a surgical trauma^38^. In addition, as the spleen is considered to be the center for regulating the immune system, splenectomy can lead to immune imbalance, with possible resultant consequences of overwhelming post-splenectomy infection, thrombosis in portal venous system, intra-abdominal abscesses/ascites, pancreatic fistulae^39,40^, and additional risks of cardiovascular complications^41^. However, the results of this study demonstrated that total splenectomy provided benefits for the immune system, and did not lead to any immune imbalance, overwhelming post-splenectomy infection, or cardiovascular complications.

Partial splenic arterial embolization (PSE) was first described by Spigos and colleagues^42^ to treat hypersplenism. It has also been used to treat portal hypertension and esophagogastric variceal bleeding^43,44^. PSE not only can increase the counts of platelets and WBCs^45–48^, but can also reduce splenic volume and improve immune function^49^. Li and collaborators^50^ reported that by maintaining the volume of PSE to 60–80% in patients with cirrhosis and hypersplenism, improved peripheral cytopenias reduced portal blood flow pressure, and less bleeding from esophagogastric varices could result. However, Kontchou and Seror^51^ argued that although PSE could be used to treat splenomegaly and hypersplenism, its indications were limited because of the possible serious complications of splenic infarction and abscesses formation which can be fatal.

Partial splenectomy is theoretically better than total splenectomy, which can preserve part of the spleen function, but there is no significant difference between the two from the actual analysis. Moreover, partial splenectomy is more difficult, longer operation time, and more bleeding after surgery.Splenectomy remains an important treatment modality in Asian countries including China and Japan^52^. Previously published studies showed that total splenectomy did not reduce humoral and cellular immunity^53^, but increased the number of blood cells and improved liver and immune functions^54^. However, not all patients with hypersplenism associated with cirrhotic portal hypertension are good candidates for total splenectomy. The indications for total splenectomy should be considered carefully for an individual patient^55^.

## Summary

The controversial issue of how total splenectomy impacts on the immune function of patients with cirrhotic portal hypertension is addressed. The results of the study showed for the first time that patients who were significantly immunocompromised before TS had their total lymphocyte counts, percentages of B lymphocytes and total T lymphocytes and their subsets to increase significantly after total splenectomy, with resultant improvements in immune function. The causes of immunocompromised status, which is of clinical importance, was discussed in this article to illustrate the indications for splenectomy in patients associated with liver cirrhosis and portal hypertension, as not all such patients should be subjected to splenectomy.

## References

[1] Bashour FN, Carlos JT, Kevin D, Mullen M (2000). Prevalence of peripheral blood cytopenias (hypersplenism) in patients with nonalcoholic liver disease. Am. Coll. Gastroeterol..

[2] Lv Y, Gong X, Xie X, Wang B, Yang Y, Li Y (2014). Clinical study on the relationship between hematocytopenia and splenomegaly caused by cirrhotic portal hypertension. Cell Biochem. Biophys..

[3] Lv Y, Lau WY, Wu H, Han X, Gong X, Liu N, Yue J, Li Q, Li Y, Deng J (2017). Causes of peripheral cytopenia in hepatitic cirrhosis and portal hypertensive splenomegaly. Exp. Biol. Med..

[4] Dameshek W (1955). Hypersplenism. Bull. N. Y. Acad. Med..

[5] Cudillo L (2009). Aplastica anemia and viral hepatitis. Mediterr. J. Hematol. Infect. Dis..

[6] Djordjevic J, Svorcan P, Vrinic D, Dapcevic B (2010). Splenomegaly and thrombocytopenia in patients with liver cirrhosis. Vojnosanit. Pregl. Military Med. Pharm. Rev..

[7] Djordjević J, Svorcan P, Vrinić D, Dapčević B (2010). Splenomegaly and thrombocytopenia in patients with liver cirrhosis. Vojnosanit Pregl..

[8] Srichaikul T, Punyagupta S, Kanchanapoom T, Chanokovat C, Likittanasombat K, Leelasiri A (2008). Hemophagocytic syndrome in dengue hemorrhagic fever with severe multiorgan complications. J. Med. Assoc. Thail. Chotmaihet Thangphaet.

[9] Barzaghi F, Passerini L, Bacchetta R (2012). Immune dysregulation, polyendocrinopathy, enteropathy, x-linked syndrome: A paradigm of immunodeficiency with autoimmunity. Front. Immunol..

[10] Zheng P, Chang X, Lu Q, Liu Y (2013). Cytopenia and autoimmune diseases: A vicious cycle fueled by mTOR dysregulation in hematopoietic stem cells. J. Autoimmun..

[11] Singh N, Yu VL, Mieles LA, Wagener MM (1993). Beta-lactam antibiotic-induced leukopenia in severe hepatic dysfunction: Risk factors and implications for dosing in patients with liver disease. Am. J. Med..

[12] Cobo F, De Celis G, Pereira A, Latorre X, Pujadas J, Albiol S (2007). Oxaliplatin-induced immune hemolytic anemia: A case report and review of the literature. Anticancer Drugs.

[13] Saif MW, Lee AM, Offer SM, McConnell K, Relias V, Diasio RB (2014). A dpyd variant (Y186C) specific to individuals of African descent in a patient with life-threatening 5-FU toxic effects: Potential for an individualized medicine approach. Mayo Clin. Proc..

[14] Cho YG, Lee JH, Kim DS, Lee HS, Choi SI (2007). Clinical usefulness of the simple technique to diagnose thrombocytopenia using immature platelet fraction. Korean J. Lab. Med..

[15] Lavigne C, Lavigne E, Massenet D, Binet C, Bremond JL, Prigent D (2005). Role of vitamin deficiency in pancytopenia in Djibouti. Findings in a series of 81 consectutive patients. Med. Trop. Rev. Corps Sante Colon..

[16] Abrishami F, Golshan A (2013). Frequency of iron deficiency anemia in girls studying in Mashhad high schools. Iran. J. Pediatr. Hematol. Oncol..

[17] Xuetao C (2013). Medical Immunology.

[18] Bashour FN, Teran JC, Mullen KD (2000). Prevalence of peripheral blood cytopenias (hypersplenism) in patients with nonalcoholic chronic liver disease. Am. J. Gastroenterol..

[19] Terai S, Takami T, Yamamoto N (2014). Status and prospects of liver cirrhosis treatment by using bone marrow-derived cells and mesenchymal cells. Tissue Eng. Part B Rev..

[20] Owman T, Lunderquist A, Alwmark A (1979). Embolization of the pleen for treatment of splenomegaly and hypersplenism in patients with portal hypertension. Investig. Radiol..

[21] Pearce EJ, Caspar P, Grzych JM, Lewis FA, Sher A (1991). Downregulation of Th1 cytokine production accompanies induction of Th2 responses by a parasitic helminth, *Schistosoma mansoni*. J. Exp. Med..

[22] Chu HB, Zhang TG, Zhao JH, Jian FG, Xu YB, Wang T, Wang M, Tang JY, Sun HJ, Li K, Guo WJ, Zhu XJ (2014). Assessment of immune cells and function of the residual spleen after subtotal splenectomy due to splenomegaly in cirrhotic patients. BMC Immunol..

[23] Eissa LA, Gad LS, Rabie AM, El-Gayar AM (2008). Thrombopoietin level in patients with chronic liver diseases. Ann. Hepatol..

[24] Wolber EM, Ganschow R, Burdelski M, Jelkmann W (1999). Hepatic thrombopoietin mRNA levels in acute and chronic liver failure of childhood. Hepatology.

[25] Ichikawa N, Kitano K, Shimodaira S, Ishida F, Ito T, Kajikawa S, Tahara T, Kato T, Kiyosawa K (1998). Changes in serum thrombopoietin levels after splenectomy. Acta Haematol..

[26] Garibaldi B, King KE, Jaffe JM, Moliterno AR (2008). Hypersplenism induced by splenic vein ligation. Am. J. Hematol..

[27] Graffner H, Gullstrand P, Hallberg T (1982). Immunocompetence after incidental aplenectomy. Scand. J. Hematol..

[28] Lv YF, Li XQ, Gong XG, Xie XH, Han XY, Wang BC (2013). Effect of surgery treatment on hypersplenism caused by cirrhotic portal hypertension. Minerva Chir.

[29] Lv Y, Lau WY, Li Y, Deng J, Dong Y, Han X, Gong X, Liu N, Wu H (2016). Hypersplenism: History and current status(Review). Exp. Ther. Med..

[30] Yan F, Li W, Chen JT, Zeng YM, Guo YW, Zhang FR, Li ZF (2006). cDNA microarray-based screening of differentially expressed genes in macrophages in the spleen of patients with portal hypertension and hypersplenism. Nan Fang Yi Ke Da Xue Xue Bao.

[31] Pham BN, Martinot-Peignoux M, Mosnier JF, Njapoum C, Marcellin P, Bougy F, Degott C, Erlinger S, Cohen JH, Degos F (1995). CD4^+^/CD8^+^ ratio of liver-derived lymphocytes is related to viraemia and not to hepatitis C virus genotypes in chronic hepatitis C. Clin. Exp. Immunol..

[32] Sato H, Adachi E, Lim LA, Koga M, Koibuchi T, Tsutsumi T, Yotsuyanagi H (2019). CD4/CD8 ratio predicts the cellular immune response to acute hepatitis C in HIV-coinfected adults. J. Infect. Chemother..

[33] Hayashi H, Takamura H, Yamaguchi Y (2012). Recent role of Hassab's operation for cirrhotic patients: Combination with endoscopic procedure for varices. Asian J. Surg..

[34] Pei Y, Chai S, Zhang Y (2019). Benefits of splenectomy and curative treatments for patients with hepatocellular carcinoma and portal hypertension: A retrospective study. Gastrointest. Surg..

[35] Kaido T, Oe H, Yoshikawa A (2004). Expressions of molecules associated with hepatocyte growth factor activation after hepatectomy in liver cirrhosis. Hepatogastroenterology.

[36] Li L, Wei W, Li Z (2018). The spleen promotes the secretion of CCL2 and supports an M1 dominant phenotype in hepatic macrophages during liver fibrosis. Cell Physiol. Biochem..

[37] Yada A, Iimuro Y, Uyama N (2015). Splenectomy attenuates murine liver fibrosis with hypersplenism stimulating hepatic accumulation of Ly-6C(lo) macrophages. J. Hepatol..

[38] Hosea SW, Brown EJ, Hamburger MI (1981). Opsonic requirements for intravascular clearance after splenectomy. N. Engl. J. Med..

[39] Tsutsumi N, Tomikawa M, Akahoshi T (2016). Pancreatic fistula after laparoscopic splenectomy in patients with hypersplenism due to liver cirrhosis: Effect of fibrin glue and polyglycolic acid felt on prophylaxis of postoperative complications. Am. J. Surg..

[40] Cheng Z, Li J, Chen J (2014). Therapeutic effects of laparoscopic splenectomy and esophagogastric devascularization on liver cirrhosis and portal hypertension in 204 cases. Laparoendosc. Adv. Surg. Tech..

[41] Weledji EP (2014). Benefits and risks of splenectomy. Int. J. Surg..

[42] Spigos DG, Jonasson O, Mozes M (1979). Partial splenic embolization in the treatment of hypersplenism. Am. J. Roentgenol..

[43] Sankararaman S, Velayuthan S, Vea R, Herbst J (2013). Severe gastric variceal bleeding successfully treated by emergency splenic artery embolization. Pediatr. Int..

[44] Huang JH, Wu PH, Gu YK, Zhang FJ, Li CX, Gao F, Zhang L, Fan WJ, Li CJ (2006). Study on primary hepatocellular carcinoma associated with hypersplenism treated by partial splenic embolization combined with hepatic arterial chemoembolization. Ai Zheng.

[45] Chikamori F, Inoue A, Okamoto H, Kuniyoshi N, Kawashima T, Takase Y (2010). Hemodynamic effects of combined therapy using partial splenic embolization and transjugular retrograde obliteration for gastric varices with gastrorenal shunt. World J. Surg..

[46] Noguchi H, Hirai K, Aoki Y, Sakata K, Tanikawa K (1995). Changes in platelet kinetics after a partial splenic arterial embolization in cirrhotic patients with hypersplenism. Hepatology.

[47] He XH, Li WT, Peng WJ, Li GD, Wang SP, Xu LC (2011). Total embolization of the main splenic artery as a supplemental treatment modality for hypersplenism. World J. Gastroenterol..

[48] Harao M, Beppu T, Masuda T, Hayashi H, Okabe H, Okabe K (2008). The significance of combined treatment for hepatocellular carcinoma with partial splenic embolization and transcatheter arterial chemoembolization using IA call/lipiodol. Gan Kagaku Ryoho Cancer Chemother..

[49] Krishnan SK, Hill A, Hillmen P, Arnold LM, Brooksbank GL, Wood A (2013). Improving cytopenia with splenic artery embolization in a patient with paroxysmal nocturnal hemoglobinuria on eculizumab. Int. J. Hematol..

[50] Zhengran L, Hong S, Kangshun Z (2002). Clinical quantitative study of therapeutic effect of partial splenic embolization (PSE) on portal vein hemodynamis. Chin. J. Radiol..

[51] N'Kontchou G, Seror O, Bourcier V, Mohand D, Ajavon Y, Castera L, Grando-Lemaire V, Ganne-Carrie N, Sellier N, Trinchet JC, Beaugrand M (2005). Partial splenic embolization in patients with cirrhosis: Efficacy, tolerance and long-term outcome in 32 patients. Eur. J. Gastroenterol. Hepatol..

[52] Yan L, Wenjing Yi, Zhansheng J, Ying Z (2015). Research progress in the treatment of hypersplenism in patients with liver cirrhosis. J. Clin. Hepatobiliary Dis..

[53] Zongjun Li (2012). The effect of splenectomy on the immune function of patients with portal hypertension complicated with hypersplenism. China Curr. Gen. Surg. Prog. Exp. Forum.

[54] Hongzhi Y (2015). The effect of splenectomy on the immune function, liver function and blood routine of patients with hepatitis B liver cirrhosis and portal hypertension combined with hypersplenism. Guangxi Med..

[55] Xiaoyu H, Ning L, Qingqing Li (2017). Summary of Hainan Provincial liver cirrhosis and portal hypertension summit seminar. Chin. Abdom. Surg..

